# Disposable and Low-Cost Electrode Based on Graphene Paper-Nafion-Bi Nanostructures for Ultra-Trace Determination of Pb(II) and Cd(II)

**DOI:** 10.3390/nano10081620

**Published:** 2020-08-18

**Authors:** Antonino Scandurra, Francesco Ruffino, Mario Urso, Maria Grazia Grimaldi, Salvo Mirabella

**Affiliations:** 1 Department of Physics and Astronomy Ettore Majorana of University of Catania, via S. Sofia 64, 95123 Catania, Italy; francesco.ruffino@ct.infn.it (F.R.); mario.urso@ct.infn.it (M.U.); mariagrazia.grimaldi@ct.infn.it (M.G.G.); salvo.mirabella@dfa.unict.it (S.M.); 2 Institute for Microelectronics and Microsystems of National Research Council of Italy (CNR-IMM), via S. Sofia 64, 95123 Catania, Italy

**Keywords:** graphene paper, perfluorosulfonic ionomer, bismuth, SWASV, trace analysis

## Abstract

There is a huge demand for rapid, reliable and low-cost methods for the analysis of heavy metals in drinking water, particularly in the range of sub-part per billion (ppb). In the present work, we describe the preparation, characterization and analytical performance of the disposable sensor to be employed in Square Wave Anodic Stripping Voltammetry (SWASV) for ultra-trace simultaneous determination of cadmium and lead. The electrode consists of graphene paper-perfluorosulfonic ionomer-bismuth nano-composite material. The electrode preparation implies a key step aimed to enhance the Bi^3+^ adsorption into nafion film, prior to the bismuth electro-deposition. Finely dispersed bismuth nanoparticles embedded in the ionomer film are obtained. The electrode was characterized by Scanning Electron Microscopy (SEM), Energy Dispersive X-ray Spectroscopy (EDX), Atomic Force Microscopy (AFM), X-ray Photoelectron Spectroscopy (XPS) and Electrochemical Impedance Spectroscopy (EIS). The electrode shows a linear response in the 5–100 ppb range, a time-stability tested up to almost three months, and detection limits up to 0.1 ppb for both Pb^2+^ and Cd^2+^. The electrode preparation method is simple and low in cost and the obtained analytical performance is very competitive with the state of art for the SWASV determination of Pb^2+^ and Cd^2+^ in solution.

## 1. Introduction

Lead and cadmium are two of the most poisonous cumulative metals that affect multiple body systems. The two metals are particularly harmful to young children because they affect cognitive functions and intellectual development [1]. Both metals are distributed in the human body to the blood, brain, liver, kidney and bones. They are stored in the teeth and bones, where they accumulate over time. Human exposure is usually assessed through the measurement of the metal concentrations in blood [2,3]. Lead in bone is released into blood during pregnancy and becomes a source of exposure to the developing fetus. There is no known level of lead exposure that is considered safe. Fortunately, lead and cadmium exposure are preventable. Cadmium possesses toxic effects on the kidneys, as well as the skeletal and respiratory systems. Moreover, it is a carcinogen [2]. Usually, natural concentrations of cadmium and lead in ground water are less than 0.5 ppb [2,3]. Nonetheless, human activity has greatly increased levels of both lead and cadmium in environmental media relevant to population exposure [2,3]. Exposures potentially of particular concern for human include, but are not limited to, mining, disposal and recycling of lead acid car batteries, ammunition, ethyl-leaded fuels, electronic and electrical waste and Ni-Cd batteries, as well as toys, jewelry and plastics containing cadmium, emissions from municipal solid waste incineration and fossil fuels. Corrosion of zinc-plated water containers, as well as soldered pipes, are additional significant sources of lead and cadmium contamination. The aforementioned contamination sources have a potential impact on drinking water as the consequence of aquifer contamination. The U.S. Environmental Protection Agency (EPA) established, in the Drinking Water Requirements for lead and cadmium, that actions have to be taken at level of 15 and 5 ppb, respectively [4]. The European Directive on the quality of water intended for human consumption established that action must be taken at the levels of 10 and 5 ppb for lead and cadmium, respectively [5]. Among the analytical techniques for the detection of heavy metals at sub-ppb level, such as inductively coupled mass spectrometry (ICP-MS) [6] or high performance liquid chromatography (HPLC) [7], square wave anodic stripping voltammetry (SWASV) is among the most powerful, being characterized by the limits of detection below the ppb level [8,9] combined with low cost of the sensors and light instrumentation, that give the possibility of on-site measurements [10]. About two decades ago, bismuth thin film electrode was proposed instead of mercury drop electrode for the alloying of heavy metals. Lu compared the sensing performance of four kinds of nanomaterials of replacing mercury that are based on metallic nanoparticles, metal oxides, carbon nanomaterials and their nanocomposites and chelating additives for analyte metal ions [8]. However, among the various kinds of nanomaterials, bismuth-based electrodes are the most promising substitutes in the analysis of lead and cadmium by SWASV for their suitable potential working window and not-poisonous properties [11,12,13,14,15]. Numerous works described the realization of electrodes and their employment by ex-situ or in-situ co-electrodeposition of bismuth and analyte metals, consisting of modified glassy carbon [16,17], graphene oxide [18], modified multiwalled carbon nanotubes [19] and screen-printed carbon paste [20,21]. Carbon-based materials used as substrate are preferable due to their advantages which include the absence of interfering metal ions, the possibility of a variety of chemical functionalization, the low cost, the high electrical conductivity and the large potential window [22,23]. In the last years, to further improve the sensitivity and quantification limit, adsorptive or hybrid adsorptive-Bi alloying based electrodes have been described in literature [24]. Nanomaterials have been used extensively in the fabrication of electrochemical sensors, due to their high surface-to-volume ratio, enhancement of mass transport and catalytic properties, are effective in improving the analytical performances [25]. In particular, graphene has the electronic and chemical properties suitable for a variety of chemical functionalization, and represents one of the most used materials for the preparation of electrodes [26]. Three-dimensional graphene–carbon nanotubes hybrid nanomaterials have been described with improved selectivity and detection limits to typical values of 0.1 and 0.2 ppb for lead and cadmium, respectively [27]. Nafion was reported in the literature for the fabrication of sensors for the detection of lead and cadmium by SWASV. The most important uses in this application concern the capping layer [28], binding material, in combination with bismuth and activated graphene on glassy carbon electrode [29,30], or forming dispersion of bismuth nanoparticles supported on the glassy carbon electrode [31]. The use of nafion alone or in combination with a chelating agent further improves the selectivity and sensitivity towards detection of cadmium and lead, with respect to bismuth-coated carbon-based materials alone. Zhao proposed a multi-walled carbon nanotube-emeraldine base polyaniline-nafion composite modified glassy carbon electrode, with a detection limit of 0.06 ppb for Cd(II) and 0.08 ppb for Pb(II) [32]. Segura proposed a glassy carbon electrode modified with nafion, 1-nitroso-2-naphthol and bismuth film for adsorptive stripping voltammetric determination of Pb(II) and Cd(II) in tap water. The detection and quantification limits were 0.08 and 0.29 ppb for Pb(II) and 0.07 and 0.24 ppb for Cd(II), respectively, with a linear detection range from 10 ppb to 70 ppb [33]. Mau Thanh proposed a glassy carbon electrode modified by in-situ co-deposition of bismuth and 8-hydroxyquinoline as chelating agent, obtaining detection limits for Pb(II), Cd(II), and Zn(II) of 0.45, 0.17 and 0.78 ppb, respectively, in the range from 2 ppb to 110 ppb [34].

However, some of the electrodes proposed in literature, despite of their very low detection and quantification limits, present weak points, the most important being related to the complexity of preparation, the consequent use of many reagents and, importantly, the expected poor stability of the active chemical species against the oxidation process. Moreover, the use of graphene in the form of flakes requires a supporting conductive material the most common being glassy carbon electrode or screen-printed carbon paste electrode. These facts lead to costly electrodes. Thus, an increasing demand for low-cost, sensitive and user friendly electrodes still exists, despite the efforts of the scientific community [8,9,10,11,12,13,14,15,16,17,18,19,20,21,22,23,24,25,26,27,28,29,30,31,32,33,34,35,36,37,38,39].

In this work, we describe a simple procedure for the preparation of high performance, stable, environmentally friendly, low-cost electrochemical electrodes for ex-situ, highly sensitive Pb^2+^ and Cd^2+^ detection in aqueous solution. The electrode consists of graphene paper, nafion and bismuth nanocomposite materials. The novelty of the proposed electrode consists of the combined use of graphene paper (which acts as a conductive path, supporting substrate and electro-active material) with nafion and bismuth electro-deposition, with a key processing step aimed at effective Bi^3+^ absorption into nafion film.

## 2. Materials and Methods

### 2.1. Materials

Sodium hydroxide, acetic acid, graphene paper 240 μm thick (XG Science) and solution of nafion 5% wt., Bi^3+^, Pb^2+^ and Cd^2+^, respectively, at 1 g dm^−3^ traceable to Standard Reference Materials (SRM) from National Institute of Standards and Technology (NIST) solutions were purchased from Sigma Aldrich, Milan, Italy. All reagents were of trace analysis grade and all aqueous solutions were made by Milli-Q water (carbon free, 18.2 MΩ × cm). Solutions of Bi^3+^, Pb^2+^ and Cd^2+^ at various concentrations were prepared by diluting the stock solutions by 0.1 M acetic buffer solution at pH 4.5.

### 2.2. Electrode Fabrication

**Figure 1:**
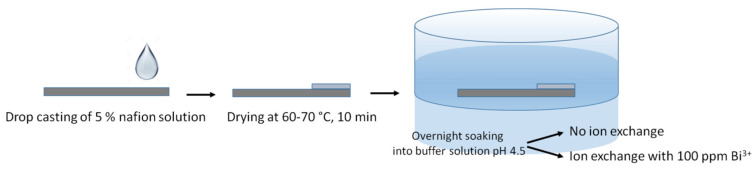
Scheme of electrodes preparation.

Strips of graphene paper 1 cm × 3 cm were used with the function of support, electrical conductor and active material of the electrode. About 15 μL of nafion solution was casted onto a portion having an area of 1 cm^2^ on both side of graphene paper and dried at 70 °C, for 10 min in air. The latter portion represents the active region of the electrode. The remaining part of the strip of graphene paper was isolated from the solution and represents the conductive path of electrode. The casted amount of nafion solution corresponds to a dried film with a thickness of about 0.5–0.7 μm. Then, a first batch of electrodes was soaked overnight into buffered solution at pH 4.5 of Bi^3+^ at concentration of 100 mg dm^−3^ (GP_naf_ex_Bi), according to the procedure reported in Figure 1.

The ion exchange method was employed to ensure a thorough exchange of the H^+^ ions of the sulfonic groups in the ionomer with Bi^3+^:

3 [R-SO_3_^−^ H^+^] + Bi^3+^ ⇄ [3(R-SO_3_^−^) Bi^3+^] + 3H^+^

Nafion is a perfluorosulfonic ionomer containing sulfonic groups at a typical concentration of about 17.5 wt.% [40]. For comparison, to study the effects of overnight ion exchange, a second batch of electrodes was prepared by overnight soaking into buffer solution without bismuth ions (GP_naf_Bi). Finally, to study the effect of nafion on the electrode analytical performance, a third batch of electrodes was prepared by Bi electro-deposition directly onto graphene paper without any further treatment (GP_Bi). All batches of electrodes underwent to bismuth electro-deposition at room temperature at −1 V vs. saturated calomel electrode (SCE) for 600 s in stirred solution at 250 rpm. These conditions were optimized experimentally and provide the best uniformity of Bi. Table 1 summarizes the fabrication steps of electrodes employed in this work.

### 2.3. Instrumental Characterization

The morphology of the electrode surface was investigated by scanning electron microscopy (SEM) and atomic force microscopy (AFM). SEM and energy dispersive X-ray (EDX) analyses were conducted by Gemini 152 field emission Carl Zeiss Supra 25. The analyses were performed at an acceleration voltage of 5 kV, with an aperture size of 30 μm, a working distance of 4–5 mm, and using an In-lens detector. The AFM analyses were performed by a Bruker-Innova microscope operating in high-amplitude mode and ultra-sharpened Si tips were used (MSNL-10 from Bruker, with anisotropic geometry, radius of curvature ~2 nm, tip height ~2.5 μm, front angle ~15°, back angle ~25°, side angle 22.5°). The Si tips were substituted as soon as a resolution lost was observed during the AFM images acquisition. The AFM images were analyzed by using the SPMLABANALYSES V7.00 software (Veeco Corp., Santa Barbara, CA, USA). The surface roughness was quantified by the Root Mean Square (RMS) parameter defined as RMS = <z(x,y)^2^>^1/2^ being z(x,y) = h(x,y)−<h(x,y)> with h(x,y) the height function and <z(x,y)> the spatial average over a planar reference surface.

X-ray Photoelectron Spectroscopy (XPS) spectra were obtained using Mg kα photon source (1253.6 eV) and a hemispherical electron analyzer VG Microtech CLAM4 equipped with a multi-channeltron detector (MCD). The binding energy scale has been corrected for surface charging up, assuming the C 1s, which originates from adventitious carbon contamination, at 284.8 eV. The electrochemical measurements were obtained at room temperature by the potentiostat Versastat 4 by Princeton Applied Research. Saturated calomel electrode (SCE) and Pt electrode were used as reference and counter electrode, respectively. A 30 mL solution was used in each measurement. Electrochemical impedance spectroscopy (EIS) characterization was done in potentiostatic mode employing a 100 ppm Bi^3+^ 0.1 M acetic buffered solution at pH 4.5, which acts as electrolyte and bismuth deposition bath.

### 2.4. SWASV Procedure

In order to get reproducible results, we firstly set a measurement protocol for SWASV test employing a heavy metal accumulation step followed by a pause before performing the SWASV. The accumulation steps were done at −1.2 V vs. SCE for 1200 s under stirring at 250 rpm both for individual Pb^2+^ as well as for simultaneous Pb^2+^ and Cd^2+^ analyses. SWASV was done after a quiet period of 30 s. All measurements were performed in acetic buffer solution 0.1 M at pH 4.5, which enhances Pb and Cd deposition and stripping response [35]. The electrodes were cleaned for successive measurements by applying a potential of −0.4 V vs. SCE for 200 s to strip residual Pb and Cd present on the electrode surface. The analytical parameters were chosen according to the literature data [27,28], whereas the instrumental parameters of SWASV were optimized experimentally (Supplementary Materials Figure S1a,b). The values of pulse height, step and frequency used in this work were 75 mV, 2 mV and 2 Hz, respectively. In the experiment, the potential is scanned over the range that comprises the peaks corresponding to the analytes. The current is measured twice during each cycle, once at the end of the forward pulse (I_F_), and again at the end of the reverse pulse (I_R_). The differential value is plotted versus the applied potential. In order to test reproducibility, each electrode was used five times and an average value is considered for the calibration, with the error bar expressed by the relative standard deviation ± σ, (Supplementary Materials Figure S2).

## 3. Results

### 3.1. Morphology Characterization

**Figure 2:**
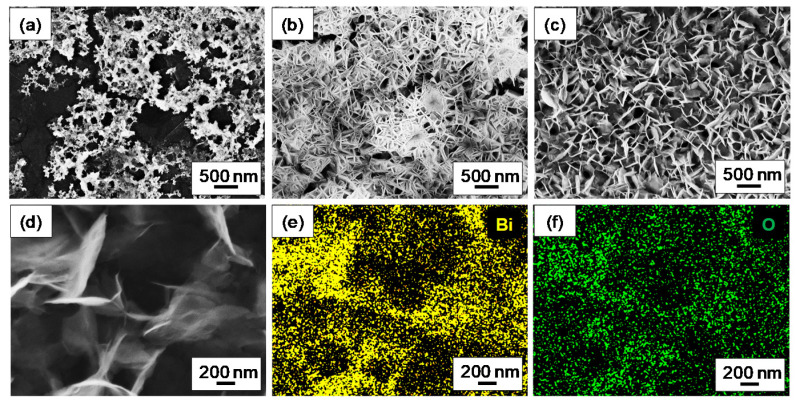
Scanning Electron Microscopy images of (**a**) GP_Bi at centre, (**b**) GP_Bi at edge of active area and (**c**) GP_naf_ex_Bi at edge of active area; (**d**) Scanning Electron Microscopy image and (**e**,**f**) corresponding chemical maps obtained by Energy Dispersive X-ray Analysis of bismuth and oxygen of GP_Bi at the edge of the active area.

**Figure 3:**
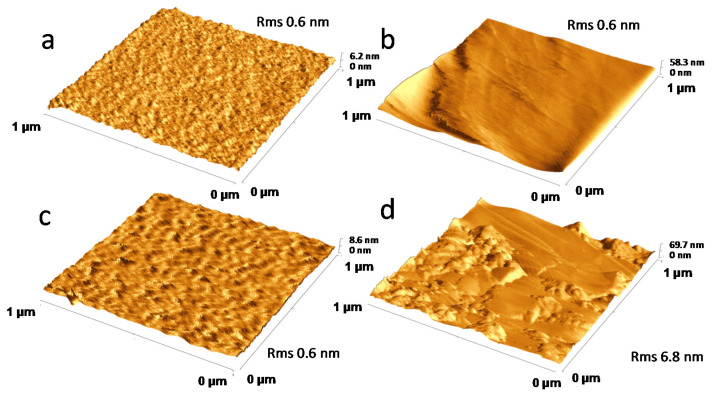
Atomic Force Microscopy images of (**a**) GP_naf_ex_Bi before bismuth electro-deposition; (**b**–**d**) GP_naf_ex_Bi after bismuth electro-deposition in three different representative regions.

Figure 2a,b report the SEM images of the GP_Bi electrode (area of 1 cm^2^) acquired in a central zone and at edge, respectively, showing a significant amount of Bi nanostructures. In the central zone, small bismuth crystals were detected. Conversely, on the edge large dendritic (or nanowalls), bismuth agglomerates were observed. The findings may be related to numerous step edges, typically present at cut edge of graphene paper, likely favoring Bi nanoparticles growth. Some authors exploited the graphite step edge to selectively grow nanoparticles by modulating the potential in a pulsed electro-deposition [36,37]. Furthermore, a possible cause of the different bismuth content observed may be related to the inhomogeneity of the potential along the large active macroscopic area of the electrode. Potential is expected to be higher on the borders, leading to an increased rate of bismuth electro-deposition. The ratio of the center to edge areas is roughly 70 to 30; both types of regions are representative of the electrode. The pictures reported in Figure 2c and in Figure S3a (Supplementary Materials) refers to the edge of the sample GP_naf_ex_Bi. In this region, once again, dendritic agglomerates of bismuth were detected. The bismuth particles are larger and more homogeneous with respect to the GP_Bi case. Figure 2d reports the SEM image of GP_Bi at edge of active area, whereas the corresponding EDX chemical maps, showing the specific contribution of bismuth and oxygen, are reported in Figure 2e,f, respectively. The presence of oxygen signal is likely owing to surface oxidation of Bi. SEM images at central area of GP_naf_ex_Bi, does not show features related to the bismuth nano-crystals, but it presents mainly a porous film of ionomer (Supplementary Materials Figure S3b). We performed AFM analyses to further characterize this region and the results are reported in Figure 3. The AFM analysis with a lateral scan of 1 μm of the GP_naf_ex_Bi before the Bi deposition (a) shows that the electrode surface is flat with a roughness characterized by a RMS of 0.6 nm. After bismuth deposition, the surface of GP_naf_ex_Bi shows three different representative regions. The first (b) is characterized by absence of significant features and by a roughness with an RMS of 0.6 nm. The second (c) shows very small structures, with a lateral size in the range of few nm, with a height of 1–1.5 nm homogeneously distributed on the surface. The roughness has a RMS 0.6 nm. The third region (d) shows aggregated structures that exhibits a lateral size of about 100 nm and height of about 10–20 nm (roughness 6.8 nm). We attributed the structures to the bismuth nanoparticles. The characterization of the GP_naf_Bi was not reported, because this electrode does not show improvements of the analytical performance (see next paragraph). The results of the SEM and AFM investigations show the presence of a high density of Bi nanoparticles whose morphology depends on the different electrode structure (presence of nafion). The ion exchange step provides fine bismuth nanoparticles embedded in the ionomer film that enhance the analytical performance (see next paragraph).

### 3.2. Analytical Performances

**Figure 4:**
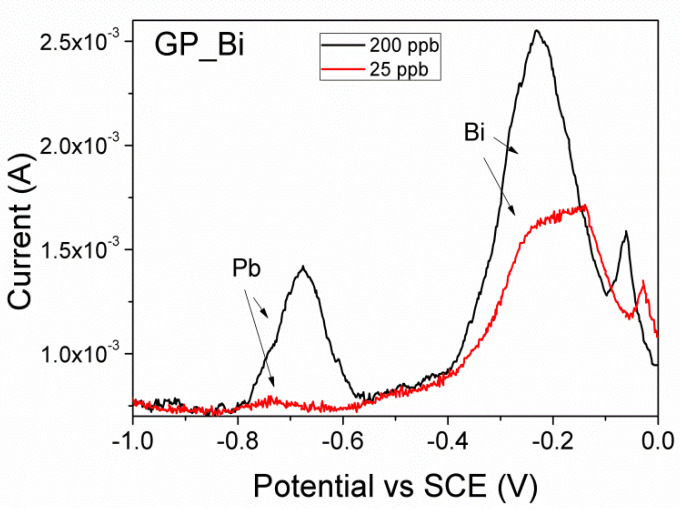
Square wave anodic stripping voltammograms of Pb^2+^ at concentrations of 200 and 25 ppb, respectively, obtained at pH 4.5, pulse height 75 mV, step 2 mV and frequency 2 Hz, by GP_Bi electrode.

Figure 4 reports typical square wave anodic stripping voltammograms obtained by GP_Bi of Pb^2+^ at concentrations of 25 (red line) and 200 (black line) ppb, respectively. The voltammograms show two peaks centered at about −0.67 and −0.22 V vs. SCE that are assigned to Pb^2+^ and Bi^3+^, respectively [14]. The anodic stripping peak corresponding to Bi^3+^ is typically broad and exhibits asymmetry which may be attributed to different Bi phases, structures, or partial bismuth oxidation. The smaller peak observed at about −0.05 V vs. SCE can be assigned to BiO_x_. Broad Bi peak was observed by Zhao using a double Bi deposition technique and MWCNT–Nafion/glassy carbon electrode [14]. The peak of Pb^2+^ at concentration of 25 ppb is slightly shifted, in comparison to 200 ppb sample, centered at about −0.73 V.

**Figure 5:**
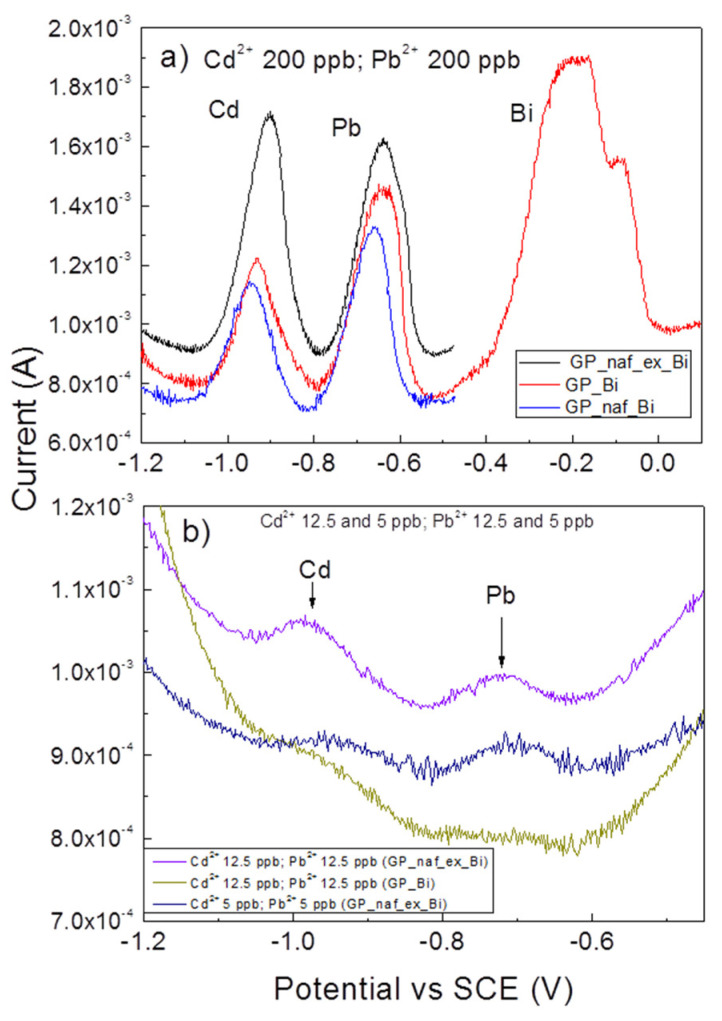
(**a**) Square wave anodic stripping voltammograms of Cd^2+^ and Pb^2+^ 200 ppb obtained by GP_naf_ex_Bi, GP_Bi and GP_naf_Bi electrodes; (**b**) square wave anodic stripping voltammograms of Cd^2+^ and Pb^2+^ 12.5 and 5 ppb obtained by GP_Bi and GP_naf_ex_Bi at pH 4.5, pulse height 75 mV, step 2 mV and frequency 2 Hz.

Afterwards, we considered the case of simultaneous detection of cadmium and lead. Figure 5a shows the anodic stripping voltammograms in the potential range from −1.2 to 0.1 V vs. SCE of simultaneous detection of Pb^2+^ 200 ppb and Cd^2+^ 200 ppb by GP_Bi (red line), GP_naf_Bi (blue line) and GP_naf_ex_Bi (black line) electrodes, respectively. Voltammograms show well-defined, intense and resolved peaks corresponding to Cd^2+^, Pb^2+^ and Bi^3+^.

The characteristic peaks of Cd^2+^ and Pb^2+^ are observed at −0.93 and −0.65 V vs. SCE for GP_Bi, at −0.95 and −0.67 V vs. SCE for GP_naf_Bi and at −0.91 and −0.63 V vs. SCE for GP_naf_ex_Bi, respectively. The values of stripping peaks of lead and cadmium are very close to that reported in literature [14,33]. The observed variations in the stripping potentials of Pb^2+^ and Cd^2+^ may be related to the variation of impedance modulus of the electrodes (as discussed later on). The GP_naf_ex_Bi electrode shows a significant increase of the response towards the Cd^2+^ detection, with respect to the other two electrodes. Conversely, the electrode GP_naf_Bi shows a reduced response of both Cd^2+^ and Pb^2+^ detection compared with the other electrodes. The explanation of this finding is not straightforward. The experimental finding may be attributed to a minor number of active sites in the GP_naf_Bi with respect to GP_naf_ex_Bi electrode. Further considerations will be discussed later on. The GP_naf_Bi electrode shows the worst analytical performance also, compared with the GP_Bi electrode, since it presents the lowest peak current intensity both for lead and cadmium stripping. Accordingly, the analytical performance of the GP_naf_Bi electrode in the concentration interval of 5–100 ppb was no longer investigated (see below). Figure 5b shows the voltammograms in the potential range from −1.2 to −0.45 V vs. SCE of simultaneous detection of Pb^2+^ and Cd^2+^ at 12.5 by GP_Bi (dark yellow line) and 12.5 (violet line) and 5 ppb (navy line) by GP_naf_ex_Bi electrodes, respectively. The peaks corresponding to Cd^2+^ and Pb^2+^ are well defined and centered at about −0.97 and −0.72 V vs. SCE, respectively. The shift of the stripping potentials of Cd^2+^ and Pb^2+^ observed at low concentrations, with respect to that at concentration of 200 ppb, occurs because the amount of deposited and, then, of stripped metals is different, according to previous studies reported by other authors [38,39].

**Figure 6:**
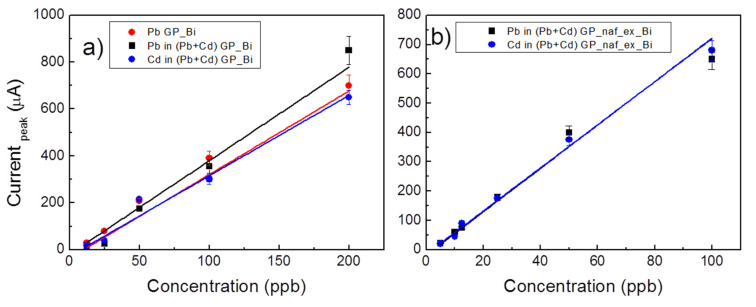
(**a**) Current calibration curves for lead and for simultaneous lead and cadmium determination by GP_Bi electrode, in the concentration range of 12.5–200 ppb; (**b**) current calibration curves for Cd^2+^ and Pb^2+^ determination by GP_naf_ex_Bi electrode in the concentration range of 5–100 ppb. Conditions: pH 4.5, pulse height 75 mV, step 2 mV and frequency 2 Hz.

Figure 6a reports the calibration curves for simultaneous determination of cadmium and lead by GP_Bi, in the concentration range of 12.5–200 ppb. The calibration curve of individual lead determination was also reported. Per each concentration, the signal was obtained by measuring from the voltammograms the anodic peaks height over a linear background (Figure 4 and Figure 5a,b) and Supplementary Materials Figures S4–S9). The simultaneous co-deposition and stripping of cadmium and lead does not worsen significantly the current responses, indicating that the analytes did not interfere with each other in the determination. The GP_Bi electrode shows a linear response in the range of 12.5–200 ppb with good linear fittings I (μA) = −19 + 3.99 c (ppb) characterized by linear correlation coefficients R^2^ of 0.983 (Pb alone), I (μA) = −35.6 + 3.56 c (ppb), R^2^ 0.814 and I (μA) = −27.4 + 3.43 c (ppb), R^2^ 0.917 for Pb^2+^ and Cd^2+^, in simultaneous determination, respectively.

The GP_naf_ex_Bi sample shows improved performance in the linear response. Calibration curves for simultaneous determination of Cd^2+^ and Pb^2+^ for this electrode are reported in Figure 6b. Linear response has been observed in the concentration range of 5–100 ppb with good linear fitting I (μA) = −14.7 + 7.36 c (ppb) with R^2^ of 0.984 and I (μA) = −18.4 + 7.41 c (ppb) with R^2^ 0.988 for Pb^2+^ and Cd^2+^, respectively. The fitting lines are very close together and not distinguishable in the plot. The slopes of the current response by GP_naf_ex_Bi electrode are higher than that observed by GP_Bi electrode. In particular, 3.56 and 3.43 μA/ppb by GP_Bi and 7.36 and 7.41 μA/ppb by GP_naf_ex_Bi for Pb^2+^ and Cd^2+^ are observed, respectively. Table 2 reports the limit of detection (LOD) and linear detection range of Cd^2+^ and Pb^2+^ by the GP_Bi and GP_naf_ex_Bi electrodes. LOD was estimated by the least square regression line method LOD = 3 × S_y/x_/b, where S_y/x_ represents the uncertainty of slope of the regression line and b is the slope of the signal/concentration functional relationship, obtained by least squares regression [41]. For comparison, Table 2 reports some of the most performing electrodes described in literature employed in SWASV and differential pulse anodic stripping voltammetry (DPASV) for the determination of zinc, cadmium and lead. Moreover, in the third column of Table 2 we reported the number of fabrication steps of the electrode employed by various authors. Accordingly, our electrodes demonstrate encouraging results, which are competitive or even better than many recent findings for Cd^2+^ and Pb^2+^ detection. In addition, our electrodes comprise few and simple preparation procedures. The best performing electrode is the GP_naf_ex_Bi, demonstrating the efficient Bi ion exchange step.

### 3.3. Electrode Stability

**Figure 7:**
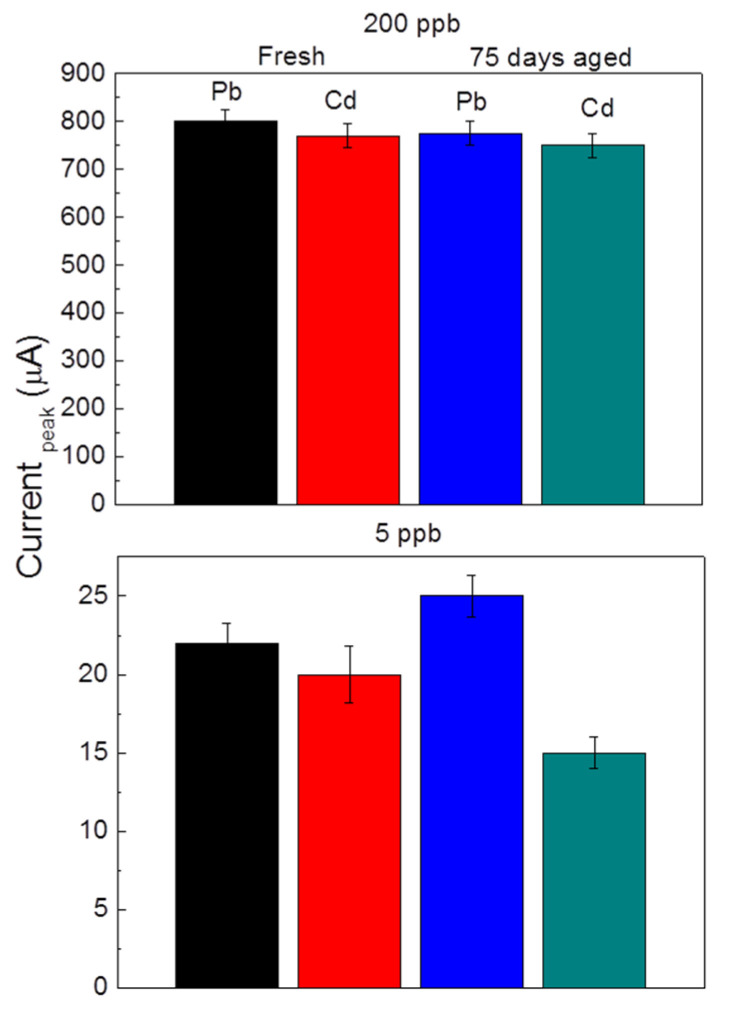
Current response for Pb^2+^ and Cd^2+^ at concentrations of 200 and 5 ppb, respectively, by fresh and 75 days aged at room temperature GP_naf_ex_Bi electrode. Conditions: pH 4.5, pulse height 75 mV, step 2 mV and frequency 2 Hz.

The stability of the GP_naf_ex_Bi electrode was evaluated by measuring the current response after 75 days from the preparation. The test has the aim to estimate the storage time at room temperature of electrodes for their reliable use. Before use, the aged electrodes were re-hydrated by soaking overnight into acetic buffer solution at pH 4.5. Alternatively, they can be stored in wet condition into a sealed sachet. Figure 7 reports the histogram of the current response for the simultaneous detection of Cd^2+^ and Pb^2+^ at the concentrations of 200 and 5 ppb, registered by fresh and 75 days stored electrodes, respectively. Little variation, always within the experimental error, in the current response was observed by the aged electrodes, indicating, however, good stability. The stability is competitive with that of some literature references, e.g., tested after only 15 days of storage at 5 °C, which retains about 96% of the initial response [28].

### 3.4. EIS Characterization

**Figure 8:**
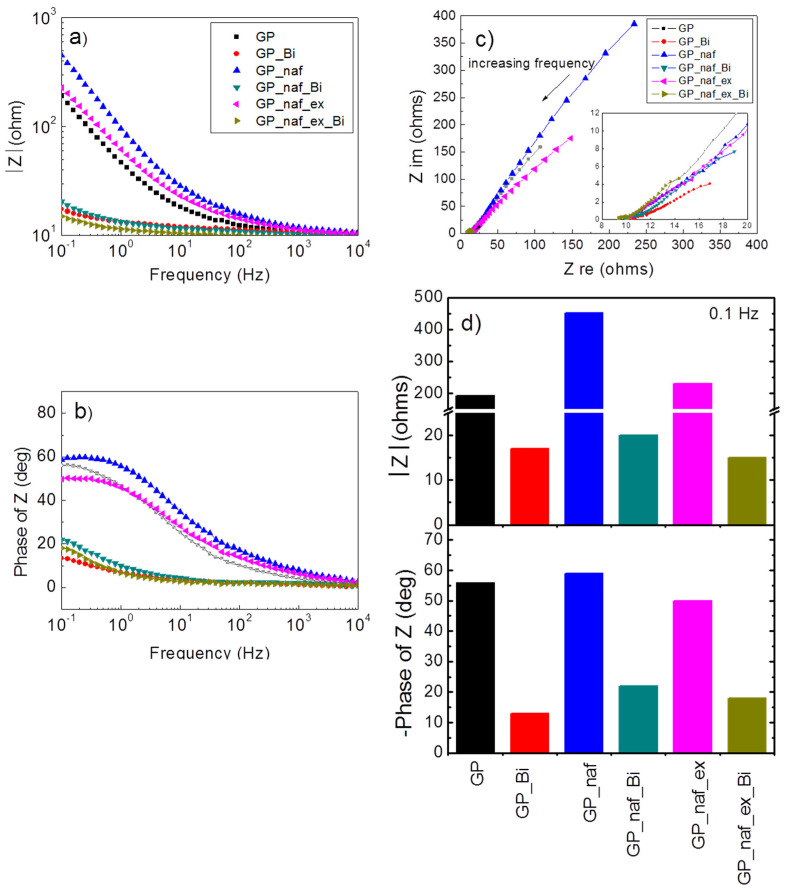
(**a**) Modulus of impedance as function of frequency for different electrodes, measured before and after Bi deposition; (**b**) corresponding phase spectra; (**c**) corresponding Nyquist plot; (**d**) impedance modulus |Z| (ohms) and phase of Z (deg) of electrodes measured at a frequency of 0.1 Hz. Solution: 100 ppm of Bi^3+^ in 0.1 M acetic buffer at pH 4.5.

In order to better investigate the role of each step in electrode preparation, EIS was performed systematically. The EIS were done before (GP, GP_naf, GP_naf_ex electrodes) and after the electro-deposition of Bi (GP_Bi, GP_naf_Bi, GP_naf_ex_Bi). Figure 8a reports the Bode plot of impedance (modulus). The impedance was measured in the frequency range of 0.1 to 10^4^ Hz. Figure 8b reports the corresponding Bode plot for the impedance phase. The presence of bismuth reduces the modulus of the impedance and values of phase in the explored frequency range, in all electrodes. Figure 8c reports the Nyquist plot of the imaginary impedance component as function of real impedance component. The plot shows that the lowest values of both Zim and Zre impedances are exhibited by the electrode GP_naf_ex_Bi (see inset in Figure 8c. According to our finding, low impedance of the electrode is expected to improve the analytical performances in SWASV, both in the analytes accumulation step, as well as increasing the stripping current.

The observed variation in the impedance modulus and phase can be related to the physico-chemical properties of the electrodes. The GP_naf_Bi electrode shows an increasing of the impedance modulus and phase with respect to GP_Bi. This may be related to the presence of ionomer layer not completely saturated by Bi^3+^/Bi°. In fact, the duration of bismuth electro-deposition (1200 s) may be insufficient for the complete diffusion of Bi^3+^ into the nafion layer. The key process of the Bi ion exchange step proposed here (GP_naf_ex_Bi electrode) produces a reduction of impedance modulus and phase, in particular at low frequencies. In this case, the overnight soaking into bismuth solution allows to the Bi^3+^ ions to completely diffuse into the ionomer layer. We reported the histogram that compares the modulus of impedance and phase measured at a frequency of 0.1 Hz in Figure 8d, to facilitate visualization. This frequency is close to that adopted in the SWASV measurements. Low electrode impedance is expected to improve the detection sensitivity, thus the proposed combination of ion exchange and Bi deposition allows an improvement of the electrode performances.

### 3.5. Surface Characterization

**Figure 9:**
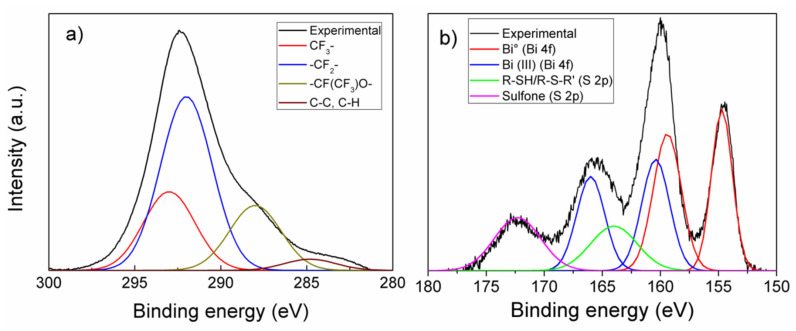
(**a**) X-ray Photoelectron Spectroscopy (XPS) C 1s core shell spectrum and (**b**) S 2p and Bi 4f spectral region of GP_naf_ex_Bi.

To better understand the chemistry of Pb and Cd detection by the GP_naf_ex_Bi electrode, some specific XPS analyses were performed. Survey XPS spectrum (not shown) exhibits the F, O, C, Na, S and Bi signals. Figure 9a shows the XPS C 1 s core shell spectrum that was deconvoluted by four Gaussian components centered at 293, 292, and 288 eV that are assigned to CF_3_–, –CF_2_–, –CF(CF_3_)O– functional groups, respectively, characteristics of the nafion structure [42,43]. The weak component centered at 284.8 eV can be attributed to adventitious carbon. Figure 9b shows the XPS spectrum in the region of 150–180 eV of binding energy, which comprises the S 2p and Bi 4f core shell signals. The spectrum was deconvoluted using four Gaussian components centered at 154.7 eV (assigned to Bi° 4f_7/2_), 159.4 eV (Bi° 4f_5/2_), 160.4 eV (Bi(III)-sulfone 4f_7/2_) and 166 eV (Bi(III)-sulfone 4f_5/2_). Moreover, two additional components at binding energy of 164 eV and 172.3 eV (each formed by not resolved S 2p_1/2_ and S 2p_3/2_ doublets) were assigned to R-SH/R-S-R’ and sulfonic group, respectively [43]. The R–SH group is formed during electrochemical reduction at the bismuth deposition step. The –SH group has a high binding affinity towards lead and cadmium ion, which acts as chelating specie, further improving the electrode sensitivity [44]. The XPS spectrum of Figure 9b suffers from differential charging up, because of the insulating nature of nafion in the dry condition and the presence of nanoparticles of metallic bismuth. As consequence of the differential charging up, the binding energy assigned to Bi° 4f_7/2_ and R–SO_3_^−^ S 2p are slightly different with respect to the literature references. The differential charging up effect, often, is a powerful tool and helps in the structural study of nanomaterials [45,46]. The observed differential charging up between the Bi 4f components assigned to Bi° and Bi(III), respectively, in the spectrum of Figure 9b, confirms the presence of bismuth in the forms of Bi(III) as well as in the metallic Bi°. Component peak areas were used to calculate the concentration of bismuth in comparison to the sulfur groups, as far as the chemical composition of the first layers can be measured relatively to the technique resolution. Atomic sensitivity factors of 9.19 and 0.668 for Bi 4f and S 2p, respectively, were used [47]. The relative composition of Bi°, Bi(III) and sulfur containing groups is reported in Table 3. The overall amount of bismuth (at.%) is about one third of the amount of sulfur containing groups.

### 3.6. Interfering Species and Application to Real Sample Analysis

The proposed electrode is intended mainly for drinking water analysis. The applicability to the real sample analysis could be, in principle, affected by the presence of interfering species. Interfering species may be due to metal ions that can compete with Pb^2+^ or Cd^2+^ for the active sites on the electrode surface. Zhao [32] identified Cu^2+^ as the most common interfering ion in the detection of Pb^2+^ and Cd^2+^ in soil analysis. However, this interfering agent can be eliminated by precipitation with ferrocyanide before Pb and Cd analysis. Segura [33], for an electrode similar to ours, found that Fe^3+^, Mo^3+^, Cu^2+^, As^3+^, Al^3+^, Zn^2+^, Co^2+^, and Ni^2+^ ions do not cause a significant interfering effect in the analysis of water real samples. Additionally, interference can also arise from species present in real samples that complex the metal ions, such as CN^−^, oxalate, NH_3_. However, the latter species are not allowed in drinking water. As good practice, it is recommended to eliminate the interfering species before performing the analysis. On the other hand, the analysis of drinking water still requires the use of pH buffer reagents (acetic acid and sodium acetate), using the standard addition method which provides the buffering of pH and also the stabilization of ion strength, limiting the matrix effects of potential interfering ions. According to previous studies performed by electrodes similar to ours [33,38], the proposed electrode can be successful applicable to the analysis of real samples of drinking water.

## 4. Conclusions

By a facile and low-cost procedure, we realized disposable electrodes, based on graphene paper, allowing ultra-trace simultaneous analysis of Cd^2+^ and Pb^2+^, by means of square wave anodic stripping voltammetry. Finely dispersed Bi nanoparticles are obtained by electro-deposition onto a nafion film deposited on graphene paper. A key Bi ion exchange step during the electrode preparation allows the reduction the impedance modulus and phase, which is associated with better performances. During the electro-deposition of bismuth, the sulfonic groups are partially reduced to –SH and organic sulfides, which further improve the electrode sensitivity, particularly towards cadmium. Three main mechanisms can be taken into account to explain the improvement in detection mechanism: (a) negative charges of sulfonic groups –SO_3_^−^ in the nafion attract the positive ions and repulse the negative counter ions; (b) the sulfonic and its reduced group thiol –SH bind the Cd^2+^ and Pb^2+^ ions increasing the accumulation; (c) finely dispersed metallic bismuth binds the reduced cadmium and lead to form metallic alloys. The electrode shows good stability and unmodified response to both Cd^2+^ and Pb^2+^ after almost three months from its preparation.

## Figures and Tables

**Table 1:** Electrodes assembly on graphene paper (GP) substrate.

Electrode Name	Nafion Layer	Bi Electro-Deposition
GP_Bi	Not present	yes
GP_naf_Bi	Overnight soaked without ion exchange	yes
GP_naf_ex_Bi	Overnight soaked with Bi^3+^ ion exchange	yes

**Table 2:** Limit of detection (LOD) of Cd^2+^ and Pb^2+^ by the GP_Bi and GP_naf_ex_Bi electrodes obtained by the least square regression line method. For comparison, some of the most performing electrodes described in literature are reported.

Analytes	Electrode Structure	Steps of Production ^(1)^	Method	LOD (ppb)		Linear Detection Range (ppb)	Reference
				**Cd** ^**2+**^	**Pb** ^**2+**^		
Pb + Cd	Bi dendritic on glassy carbon H_2_ template	2	SWASV	0.4	0.1	5–50	[38]
Pb + Cd + Zn	Nafion coated Bi electrode	5	DPASV	0.17	0.17	4–36	[29]
Pb + Cd	Mesoporous graphene	5	DPASV	0.5	0.1	0.5–110	[18]
Pb + Cd	MWCNT + graphene oxide	16	DPASV	0.1	0.2	0.5–30	[27]
Pb + Cd	Bi PSS composite	5		0.1	0.27	5–40	[20]
Pb + Cd	Graphene oxide + nafion + BMIM-PF₆ ^(2)^	7	SWASV	0.33	0.42	2.4–70	[28]
Pb	Bi electro-deposition on graphene paper	1	SWASV	-	0.15	12.5–200	This work
Pb + Cd	Bi electro-deposition on graphene paper	1	SWASV	0.25	0.12	12.5–200	This work
Pb + Cd	nafion deposition on graphene paper, Bi^3+^ ion exchange + Bi electro-deposition	3	SWASV	0.1	0.1	5–100	This work

^(1)^ graphene paper, graphene or graphene oxide preparation steps are not included. ^(2)^ 1-Butyl-3-methylimidazolium hexafluorophosphate.

**Table 3:** Elemental composition (atomic concentration %) corresponding to Bi°, Bi(III), R–SO_3_^−^ and R-SH/R-S-R’ of GP_naf_ex_Bi electrode.

	Bi°	Bi(III)	R-SO_3_^−^	R-SH/R-S-R’
GP_naf_ex_Bi	16.2	11.2	39.5	33.1

## Supplementary Materials

The following are available online at https://www.mdpi.com/2079-4991/10/8/1620/s1, Figure S1: (a) effect of pulse height and (b) of frequency on the shape and intensity of the anodic tripping peak of lead at pH 4.5; Figure S2: Procedure used to measure the height of stripping peak current from the SWAS voltammograms; Figure S3: (a) high resolution SEM of the edge of the GP_naf_ex_Bi electrode; (b) SEM picture of the GP_naf_ex_Bi electrode in the centre of active area. The surface morphology is characterized by significant porosity which is useful to improve the electrode sensitivity; Figure S4: SWAS voltammograms of Pb(II) at 25 and 12.5 ppb detected by the GP_Bi electrode Conditions: pH 4.5, pulse height 75 mV, step 2 mV and frequency 2 Hz; Figure S5: Pb(II) + Cd(II) at 200, 100 and 50 ppb detected by the GP_Bi electrode Conditions: pH 4.5, pulse height 75 mV, step 2 mV and frequency 2 Hz; Figure S6: SWAS voltammograms of Pb(II) + Cd(II) at 50, 25, 12.5 ppb detected by the GP_Bi electrode Conditions: pH 4.5, pulse height 75 mV, step 2 mV and frequency 2 Hz; Figure S7: SWAS voltammograms of Pb(II) + Cd(II) at 25 and 12.5 ppb scanned up to −0.4 V vs. SCE. The peak of bismuth is not visible in this range. Conditions: pH 4.5, pulse height 75 mV, step 2 mV and frequency 2 Hz; Figure S8: SWAS voltammograms of Pb(II) + Cd(II) at 200, 100 and 50 ppb detected by GP_naf_ex_Bi electrode. The signals, in particular of Cd(II), are significant higher than that obtained by GP_Bi electrode. Conditions: pH 4.5, pulse height 75 mV, step 2 mV and frequency 2 Hz; Figure S9: SWAS voltammograms of Pb(II) + Cd(II) at 12.5 ppb detected by GP_naf_ex_Bi electrode. The scanned potential comprises the Bi stripping peak. Conditions: pH 4.5, pulse height 75 mV, step 2 mV and frequency 2 Hz.
