# Supplementary material for: Disposable and Low-Cost Electrode Based on Graphene Paper-Nafion-Bi Nanostructures for Ultra-Trace Determination of Pb(II) and Cd(II)
Source: Nanomaterials (Basel). 2020 Aug 18;10(8):1620. doi: 10.3390/nano10081620 (PMC7466693; doi:10.3390/nano10081620)
Supplement: Supplementary file 1 [file nanomaterials-10-01620-s001.pdf]

nanomaterials

Supplementary materials

Disposable and Low-Cost Electrode Based on Graphene Paper-Nafion-Bi Nanostructures for Ultra-Trace Determination of Pb(II) and Cd(II)

Antonino Scandurra [1,*], Francesco Ruffino [1,2], Mario Urso [1], Maria Grazia Grimaldi [1,2] and Salvo Mirabella [1,2]

1 Department of Physics and Astronomy Ettore Majorana of University of Catania, via S. Sofia 64, 95123 Catania, Italy; francesco.ruffino@ct.infn.it (F.R.); mario.urso@ct.infn.it (M.U.); mariagrazia.grimaldi@ct.infn.it (M.G.G.); salvo.mirabella@dfa.unict.it (S.M.)

2 Institute for Microelectronics and Microsystems of National Research Council of Italy (CNR-IMM), via S. Sofia 64, 95123 Catania, Italy

* Correspondence: ascandurra@unict.it; Tel.: +39-095-3785289

Received: 15 July 2020; Accepted: 13 August 2020; Published: date

Instrumental parameters optimization of SWASV

The instrumental parameters of SWASV are pulse height, frequency (or pulse period) and step increment of potential. The parameters have been optimized to obtain anodic stripping peak with highest symmetry and lowest full width at half maximum (FWHM). Figures 1a and 1b report the effect of pulse height and frequency, respectively, studied by a Pb^{2+} 100 ppm buffered solution at pH 4.5. Pulse height was explored in the range of 25–100 mV at constant step increment of 2 mV and frequency of 10 Hz. The peak current intensity increases significantly with the pulse height. The frequency was explored in the range of 2–25 Hz at constant pulse height and step increment of 75 and 2 mV, respectively. The best peak shape was obtained at a frequency of 2 Hz which corresponds to a pulse period of 0.5 s. The step increment was explored in the range of 5–20 mV. The best combination of parameters was pulse height 75 mV, frequency 2 Hz and step increment of 2 mV which corresponds to a scan rate of 4 mV s^{-1}. A FWHM of about 100 mV was obtained for the peak of lead. The selected parameters values are closest to that used by other authors [1–3].

a) *b)*

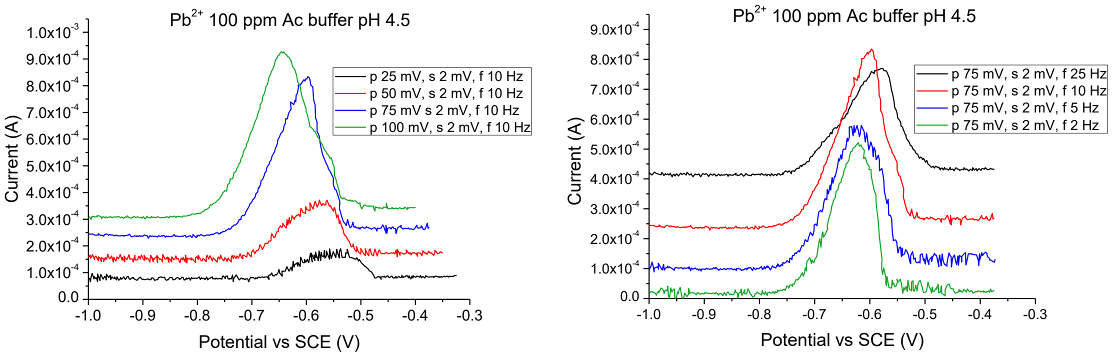

Figure S1. (**a**) SWAS voltammograms of Pb^{2+} at pH 4.5 as function of pulse height and (**b**) of frequency on the shape and intensity of the anodic tripping peak of lead at pH 4.5.

Construction of the calibration curves and estimation of limit of detection (LOD)

Calibration curves of lead and simultaneous lead and cadmium quantitative determinations were constructed starting from the voltammograms, acquired at various analytes concentration (Figures 4, 5 (**a**)–(**b**) and additional Figures **S4–S9**). The stripping peak currents were measured by baseline subtraction, as depicted in the example reported in Figure **S2**.

LODs for lead and cadmium were obtained by the standard error of estimate, $S_{y/x}$. The approach based on the standard error of the estimate, the statistic $S_{y/x}$, avoids the measurements of the signal of blank samples. The value of $S_{y/x}$, is calculated when performing ordinary least square regression (OLS), which allows the evaluation of the uncertainty of slope and intercept of the regression line. Then, we calculated the LOD as follows:

$LOD = 3 \times S_{y/x} / b$

where b is the slope of the signal/concentration functional relationship, usually obtained by ordinary least squares regression (OLS) [4].

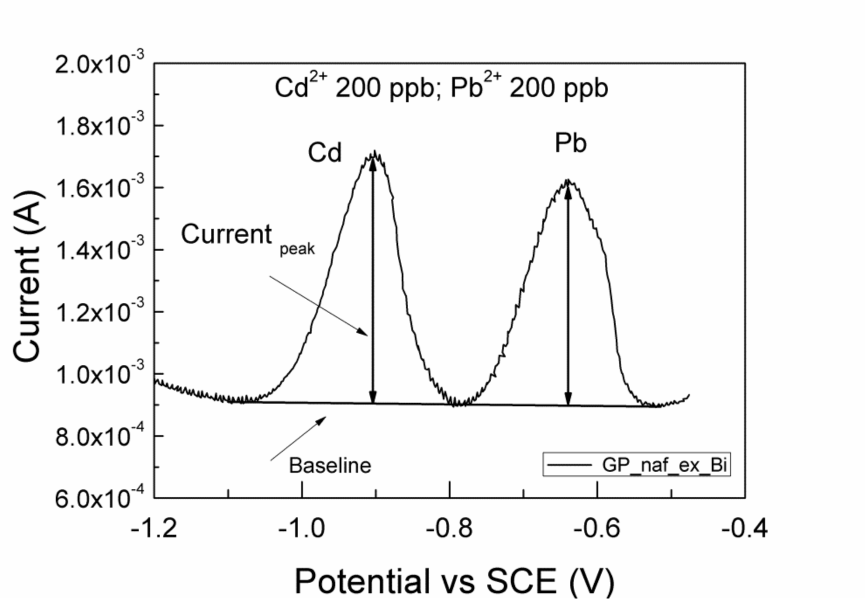

Figure S2. Procedure used to measure the height of stripping peak current from the SWAS voltammograms.

.

SEM of GP_naf_ex_Bi in the edge and centre of active area

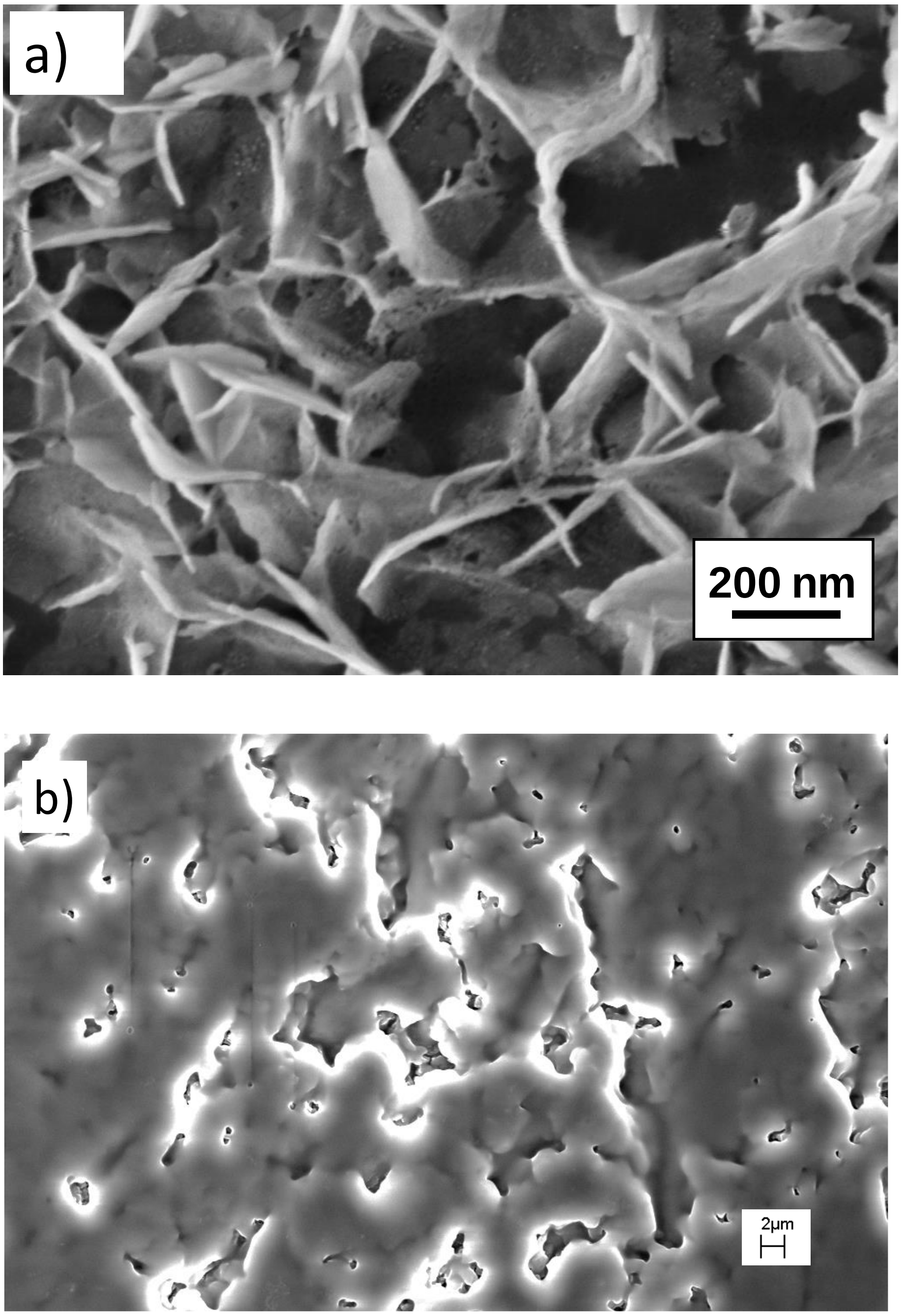

Figure S3. (**a**) high resolution SEM of the edge of the GP_naf_ex_Bi electrode; (**b**) SEM picture of the GP_naf_ex_Bi electrode in the centre of active area. The surface morphology is characterized by significant porosity which is useful to improve the electrode sensitivity.

Typical SWAS voltammograms (raw data) acquired in ultra trace analysis of Pb(II) and Cd(II)

Pb(II) by GP_Bi

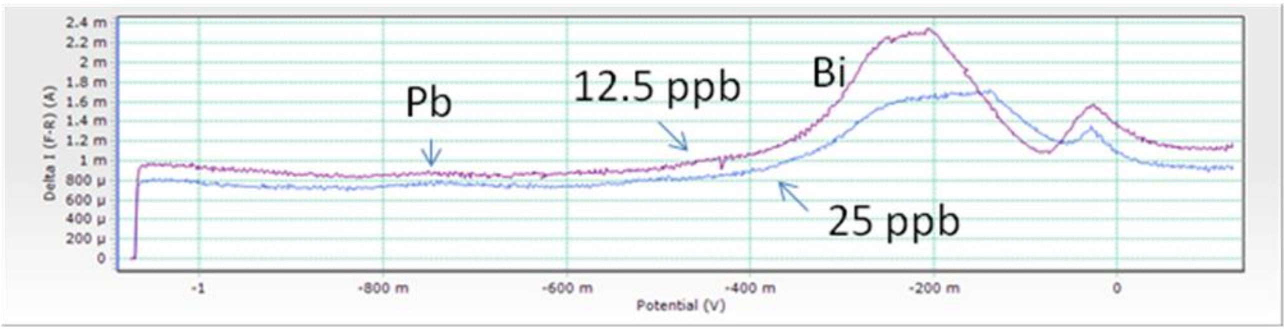

Figure S4. SWAS voltammograms of Pb(II) at 25 and 12.5 ppb detected by the GP_Bi electrode. Conditions: pH 4.5, pulse height 75 mV, step 2 mV and frequency 2 Hz.

Pb(II) + Cd(II) by GP_Bi

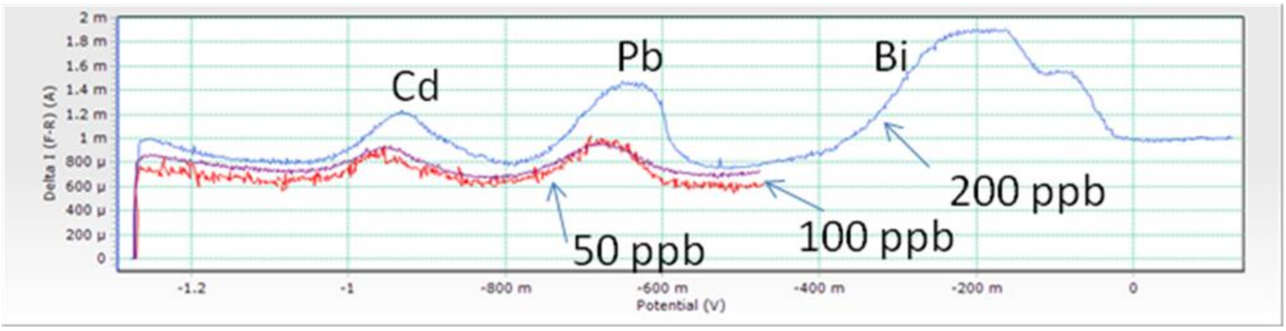

Figure S5. SWAS voltammograms of Pb(II) + Cd(II) at 200, 100 and 50 ppb detected by the GP_Bi electrode. Conditions: pH 4.5, pulse height 75 mV, step 2 mV and frequency 2 Hz.

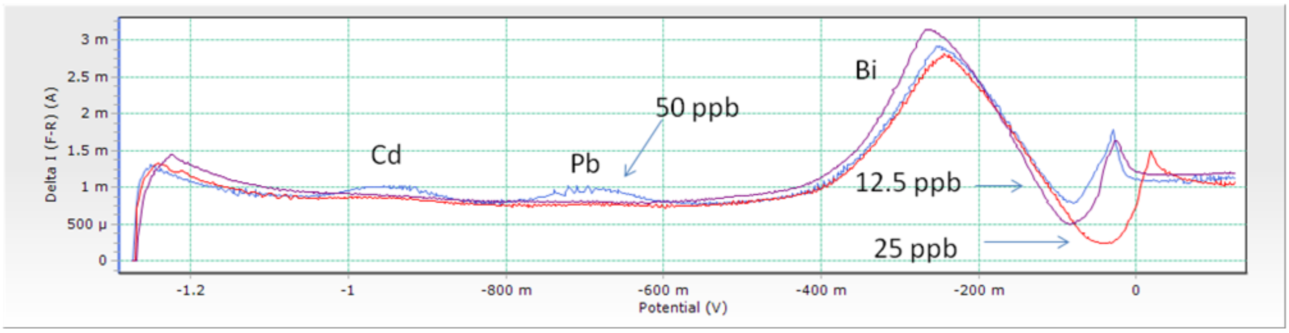

Figure S6. SWAS voltammograms of Pb(II) + Cd(II) at 50, 25, 12.5 ppb detected by the GP_Bi electrode. Conditions: pH 4.5, pulse height 75 mV, step 2 mV and frequency 2 Hz.

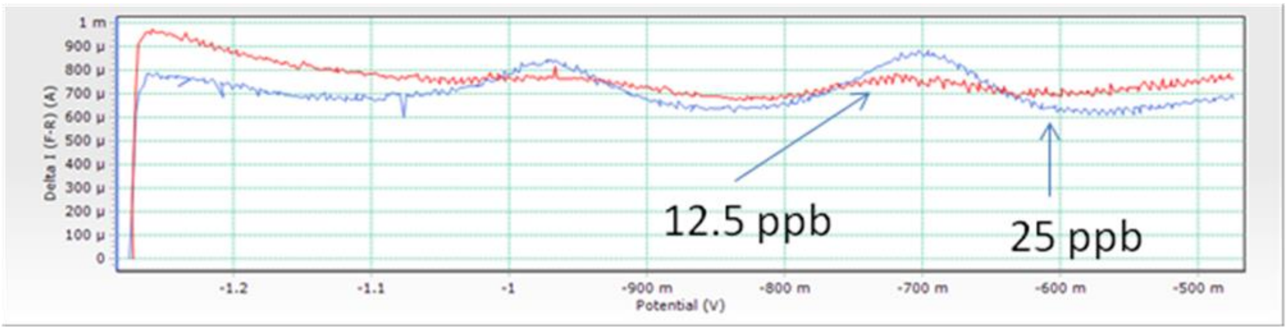

Figure S7. SWAS voltammograms of Pb(II) + Cd(II) at 25 and 12.5 ppb scanned up to -0.4 V vs SCE. The peak of bismuth is not visible in this range. Conditions: pH 4.5, pulse height 75 mV, step 2 mV and frequency 2 Hz.

Pb(II) + Cd(II) by GP_naf_ex_Bi

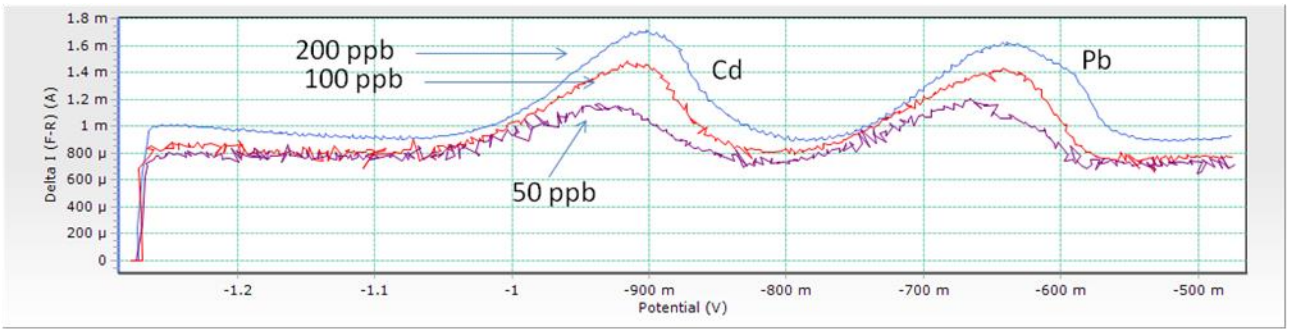

Figure S8. SWAS voltammograms of Pb(II) + Cd(II) at 200, 100 and 50 ppb detected by GP_naf_ex_Bi electrode. The signals, in particular of Cd(II), are significant higher than that obtained by GP_Bi electrode. Conditions: pH 4.5, pulse height 75 mV, step 2 mV and frequency 2 Hz.

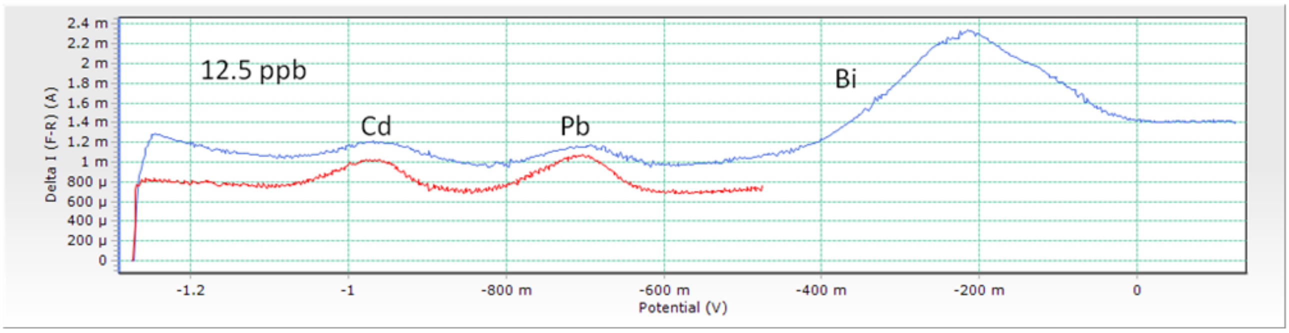

Figure S9. SWAS voltammograms of Pb(II) + Cd(II) at 12.5 ppb detected by GP_naf_ex_Bi electrode. The scanned potential comprises the Bi stripping peak. Conditions: pH 4.5, pulse height 75 mV, step 2 mV and frequency 2 Hz

References

1) Huang H., Chen T., Ma X. L. H., Ultrasensitive and simultaneous detection of heavy metal ions based on three-dimensional graphene-carbon nanotubes hybrid electrode materials, Anal. Chim. Acta 852, (2014), 45–54, http://dx.doi.org/10.1016/j.aca.2014.09.010.
2) Pandey S. K., Sachan S., Singh S. K., Ultra-trace sensing of cadmium and lead by square wave anodic stripping voltammetry using ionic liquid modified graphene oxide, Mat. Sci. Energy Technol. 2, (2019), 667–675.
3) Baldrianova L., Agrafiotou P., Svancara I., Jannakoudakis A.D., Sotiropoulos S., The effect of acetate concentration, solution pH and conductivity on the anodic stripping voltammetry of lead and cadmium ions at in situ bismuth-plated carbon microelectrodes, J. Electroanal. Chem. 660, (2011), 31–36, doi:10.1016/j.jelechem.2011.05.028.
4) Desimoni E. and Brunetti B., About Estimating the Limit of Detection by the Signal to Noise Approach, Pharm Anal Acta, 6, (2015), 355, DOI: 10.4172/2153-2435.1000355.

© 2020 by the authors. Submitted for possible open access publication under the terms and conditions of the Creative Commons Attribution (CC BY) license (http://creativecommons.org/licenses/by/4.0/).
